# Cancer-associated fibroblasts promote the stemness and progression of renal cell carcinoma via exosomal miR-181d-5p

**DOI:** 10.1038/s41420-022-01219-7

**Published:** 2022-11-01

**Authors:** Meng Ding, Xiaozhi Zhao, Xiaoqing Chen, Wenli Diao, Yansheng Kan, Wenmin Cao, Wei Chen, Bo Jiang, Haixiang Qin, Jie Gao, Junlong Zhuang, Qing Zhang, Hongqian Guo

**Affiliations:** 1 Nanjing Drum Tower Hospital Clinical College of Nanjing University of Chinese Medicine; Department of Urology, Drum Tower Hospital, Medical School of Nanjing University; Institute of Urology, Nanjing University, Nanjing, 210008 Jiangsu P.R. China; 2 State Key Laboratory of Pharmaceutical Biotechnology, School of Life Sciences, Nanjing University, Nanjing, 210046 Jiangsu P.R. China

**Keywords:** Renal cancer, Cancer microenvironment

## Abstract

The mechanisms underlying the effects of cancer-associated fibroblasts (CAFs) on cancer stemness and tumor progression in renal cell carcinoma (RCC) have not been elucidated yet. In the present study, we found that the enrichment of CAFs was positively associated with tumor progression and cancer stemness in RCC. Further investigation revealed that CAFs could enhance cancer stemness through delivering exosomes to RCC cells, and miR-181d-5p was identified as the critical exosomal miRNA in CAF-secreted exosomes by small RNA sequencing and subsequent screening assays. Mechanistically, exosomal miR-181d-5p transferred from CAFs to RCC cells directly suppressed the expression of ring finger protein 43 (RNF43) and activated Wnt/β-catenin signaling pathway, thus promoted cancer stemness and tumor progression. Overexpression of RNF43 strongly suppressed stemness properties and the effects could be reverted by miR-181d-5p. Overall, our findings revealed a crucial mechanism by which CAF-secreted exosomal miRNAs to enhance cancer stemness and thus promote RCC progression, suggesting a new avenue based on CAF-secreted miRNAs for more effective targeted therapies.

## Introduction

Renal cell carcinoma (RCC) is one of the most common types of malignant tumor, with more than 400,000 individuals worldwide per year [1]. Although RCC can be treated surgically in early stage, up to a third of patients were diagnosed with or further developed metastases, with poor clinical prognosis [2, 3]. A better understanding of the underlying mechanism of RCC remains priority for improving these issues. Currently, there are increasing studies showing that the cellular communications between cancer cells and other stroma cells of tumor microenvironment (TME) perform crucial functions in tumor development and therapy response [4, 5]. Cancer-associated fibroblasts (CAFs) constitute the majority of the stromal components in a variety of cancers, and therefore attract wide attention [6, 7]. In the past years, a burst of knowledge on how CAFs remodel the extracellular matrix (ECM) structure and interact with tumor cells or other stromal cells have suggested that CAFs may hold the key to curing malignancy [7–9]. Nevertheless, the functional importance and the detailed molecular mechanism underlying the communication between CAFs and cancer cells in RCC are yet to be revealed.

Recently, cancer stem cells (CSCs), a novel population of cancer cells possessing stem cell characteristics, are considered to be of great ability to self-renew and resist chemotherapies, thereby sustaining tumor growth and relapse [10, 11]. Similar to other normal stem cells, the sustainability of cancer “stemness” requires a supportive niche and is simultaneously regulated by intrinsic and extrinsic factors [12]. CAFs are enriched in the niches of CSCs, and have been reported to interact with CSCs to regulate their stem cell features [8, 13]. Although the promotion role of CAFs in cancer stemness has been supported by increasing studies, and multiple CAFs targeting strategies have been proposed for precise anti-CSCs treatment, the definite effect of CAFs on CSCs in different cancer types is controversial, and the underlying mechanism is still remaining to exposure [6, 7].

Exosomes are extracellular vesicles released in response to cell activation, hypoxia, injury, and cellular stress, with diameters ranging from 30 to 150 nm [14]. Exosomes contain diverse cargos, such as DNA, RNA, proteins, and lipids, that can be trafficked between cells and modulate the function of recipient cells, thus exosomes have been proposed to be novel essential mediators of cell-to-cell communication in TME and distant microenvironments [15]. MicroRNAs (miRNAs) are small noncoding RNAs with lengths of 21–23 nucleotides, and regulate almost all biological processes by promoting target mRNA degradation or translational repression [16]. Exosome-mediated miRNAs delivery is widely believed to make a huge contribution to cell-to-cell communication, and exosomal miRNAs are also crucial means for CAFs to function in TME [14], however, the function of CAFs-secreted exosomal miRNAs in RCC are rarely reported.

Herein, we intended to explore the effect of CAFs on cancer stemness and RCC progression, and focused on the molecular mechanism underlying which in terms of exosomal miRNAs. We found that the enrichment of CAFs was positively associated with cancer stemness and RCC progression by IHC staining of RCC tissue microarray chips, and then identified the crucial CAFs-delivered exosomal miR-181d-5p through small RNA sequencing and subsequent screening assays. Moreover, further experiments were also performed to illuminate the function and mechanism of exosomal miR-181d-5p in cancer stemness and RCC progression.

## Results

### The enrichment of CAFs is associated with cancer stemness and tumor progression in RCC

**Fig. 1:**
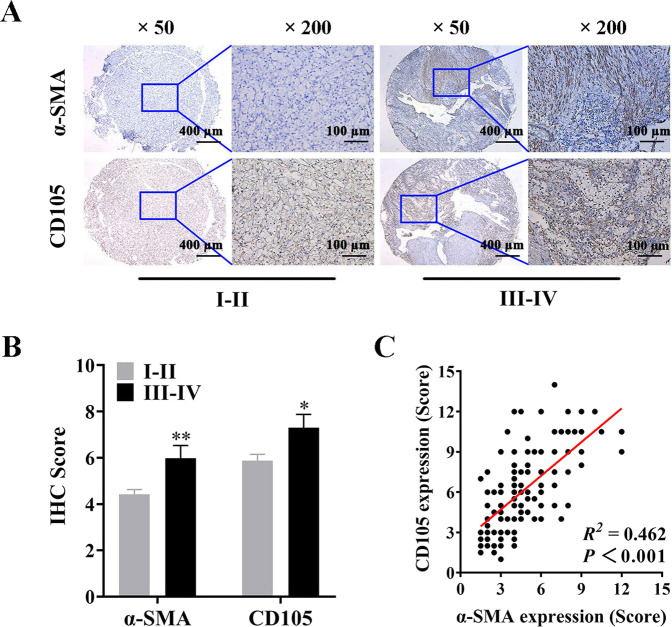
The enrichment of CAFs is associated with tumor progression and cancer stemness in RCC. **A** Representative images of IHC staining of α-SMA and CD105 in RCC tissues (*n* = 141). **B** IHC score of α-SMA and CD105 in low stage (I–II) (*n* = 111) and high stage (III–IV) (*n* = 30), the data are shown as the mean ± SEM; **C** Pearson’s correlation scatter plot of IHC score of α-SMA and CD105 (*n* = 141). *P* value between two groups was obtained by Unpaired *t* test. *P < 0.05; **P < 0.01.

CAFs are mainly involved in TME, and play critical roles in tumor development. Whereas, the function and underlying mechanism of CAFs in RCC are needed to be revealed. Cancer stemness has recently been considered as the driving force of tumorigenesis, and the maintenance mechanism of which is uncertain. To determine the relationship between CAFs and cancer stemness in clinical RCC tissues, the TMA chips contained a total of 141 case RCC samples were detected by IHC staining using specific anti-alpha-smooth muscle actin (α-SMA) and anti-endoglin (CD105) antibodies. α-SMA was identified as a specific marker of CAFs [17], and CD105-positive RCC cells were reported to possess self-renewal ability and demarcated as a cancer stem cell subpopulation [18]. The IHC staining of TMA chips showed that α-SMA and CD105 were significantly overexpressed in high-stage RCC tissues (stage III–IV) compared with low-stage RCC tissues (stage I–II) (Fig. 1A, B). Notably, Pearson’s correlation analysis showed a good relevance between the expression of α-SMA and CD105 (*R*^*2*^ = 0.462, *P* < 0.001; Fig. 1C). The detailed information on patient characteristics and IHC score of α-SMA and CD105 were shown in Supplementary Table 1. These results suggested that the enrichment of CAFs in TME might have a close relation with cancer stemness, and thus contribute to RCC progression.

### CAFs promote cancer stemness by delivering exosomes to RCC cells

**Fig. 2:**
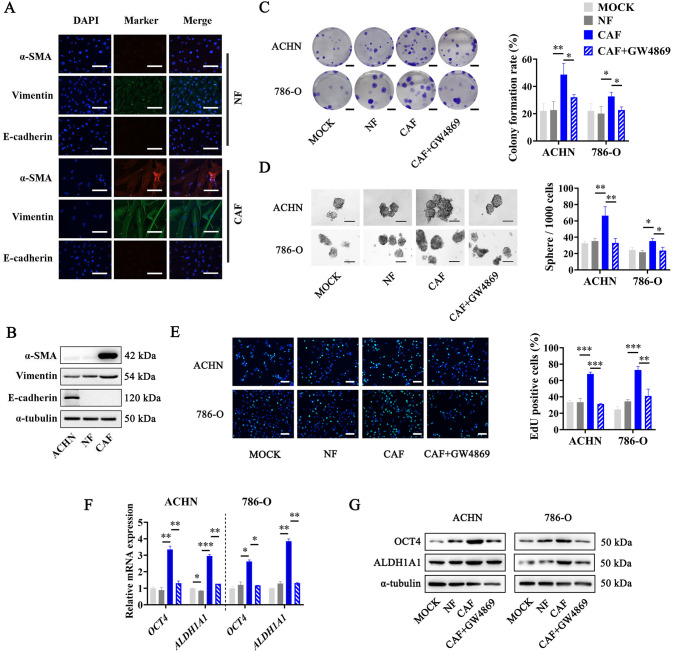
CAFs promote the cancer stemness of RCC cells through secreting exosomes. **A** Immunofluorescence staining of α-SMA, Vimentin, and E-cadherin in CAFs and NFs, scale bar = 50 µm. **B** Western Blot analysis of α-SMA, Vimentin, and E-cadherin protein levels in CAFs, NFs, and ACHN cell line. **C** Representative images (Left) and histogram statistics (Right) from colony formation assay of RCC cells co-cultured with NFs, CAFs, or GW4968 pre-treated CAFs, scale bar = 0.5 cm. **D** Representative images (Left) and histogram statistics (Right) from sphere formation assay of RCC cells co-cultured with NFs, CAFs, or GW4968 pre-treated CAFs. **E** Representative images (Left) and histogram statistics (Right) from EdU assay of RCC cells co-cultured with NFs, CAFs, or GW4968 pre-treated CAFs, scale bar = 50 µm. **F** RT-qPCR analysis of cancer stemness relative genes *OCT4* and *ALDH1A1* expression in RCC cells co-cultured with NFs, CAFs, or GW4968 pre-treated CAFs, normalized to *β-actin*. **G** Western blot of OCT4 and ALDH1A1 expression in RCC cells co-cultured with NFs, CAFs, or GW4968 pre-treated CAFs, normalized to α-Tubulin. Cell experiment was repeated three times independently, *P* value between the two groups was obtained by Unpaired *t* test. *P < 0.05; **P < 0.01; ***P < 0.001.

To confirm the effects of CAFs on RCC stemness in vitro, we firstly isolated primary NFs and CAFs from fresh RCC tissues and paired normal kidney tissues, respectively. Immunofluorescence assays and Western Blot assays were carried out to confirm the characteristic markers of CAFs and NFs. As shown in Fig. 2A, B, isolated NFs and CAFs were all positive for Vimentin, the marker of interstitial cells, but negative for E-cadherin, the marker of epithelial cells. Moreover, CAFs are enriched with α-SMA, while NFs are not. We then co-cultured NFs or CAFs with RCC cells using a 0.4 µm hanging cell culture insert, and carried out sphere formation, colony formation, and EdU assays to evaluate the effect of NFs and CAFs on RCC cells. The results showed that compared with cells co-cultured with NFs, all the sphere formation, colony formation, and proliferation abilities of ACHN and 786-O cells co-cultured with CAFs increased significantly (Fig. 2C–E). In terms of intracellular molecular signaling, OCT4 and ALDH1A1 were proved to be the key functional proteins in cancer stem cells, and could be used as cancer stemness markers of RCC cells [19, 20]. Therefore, we further investigated the mRNA and protein levels of OCT4 and ALDH1A1 in RCC cells co-cultured with NFs or CAFs, and found that RCC cells co-cultured with CAFs showed increased OCT4 and ALDH1A1 levels than cells co-cultured with NFs (Fig. 2F, G). These results revealed that CAFs could promote the cancer stemness of RCC cells.

Recent evidence demonstrated that exosomes could be secreted by kinds of cell types, including CAFs, and played a pivotal role in TME communication [21]. We conjectured that CAFs might deliver exosomes to affect cancer cells. To investigate whether CAFs sustain the stemness of RCC cells via exosomes, we pre-treated CAFs with GW4869, an exosome production inhibitor. As shown in Fig. 2C–E, pre-treating CAFs with GW4869 could greatly block the promotion effect of CAFs on the sphere formation, colony formation, and proliferation abilities of RCC cells. Furthermore, cells co-cultured with GW4869 pre-treated CAFs showed significantly lower expression levels of OCT4 and ALDH1A1 than cells co-cultured with CAFs (Fig. 2F, G). These results suggested a critical role of CAFs-delivered exosomes in the promotion of RCC stemness.

**Fig. 3:**
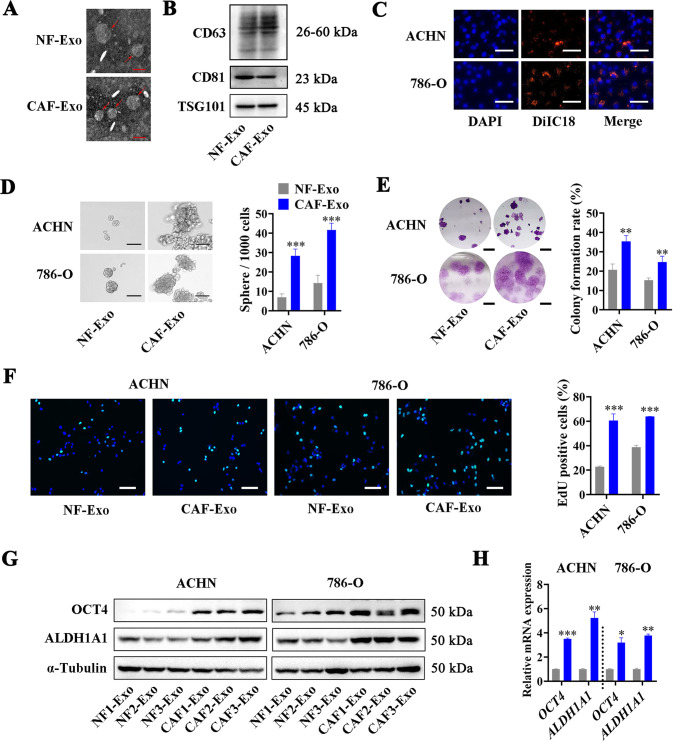
CAF-derived exosomes promote the cancer stemness of RCC cells. **A** Representative images of transmission electron micrograph of NFs-secreted exosomes (NF-Exo) and CAFs-secreted exosomes (CAF-Exo), scale bar = 100 nm. **B** Western blot analysis of exosome markers CD63, CD81, and TSG101 in NF-Exo and CAF-Exo. **C** Transfer of exosomes from CAFs to RCC cells examined by confocal microscope, scale bar = 50 µm. **D** Representative images (Left) and histogram statistics (Right) from sphere formation assay of NF-Exo or CAF-Exo treated RCC cells, scale bar = 100 µm. **E** Representative images (Left) and histogram statistics (Right) from colony formation assay of NF-Exo or CAF-Exo treated RCC cells, scale bar = 0.5 cm. **F** Representative images (Left) and histogram statistics (Right) from EdU assay of NF-Exo or CAF-Exo treated RCC cells, scale bar = 50 µm. **G** Western Blot analysis of OCT4 and ALDH1A1 expression in NF-Exo or CAF-Exo treated RCC cells, normalized to α-Tubulin. **H** RT-qPCR analysis of *OCT4* and *ALDH1A1* expression in NF-Exo or CAF-Exo treated RCC cells, normalized to *β-actin*. Cell experiment was repeated three times independently, *P* value between two groups was obtained by Unpaired *t* test. *P < 0.05; **P < 0.01; ***P < 0.001.

To further directly demonstrate the function of CAFs-delivered exosomes. we isolated exosomes from a conditioned medium of CAFs (CAF-Exo) and NFs (NF-Exo). Transmission electron microscopy and NanoSight analysis confirmed the size and shape of the isolated exosomes, which were typically cup-shaped, and with size of 50–150 nm in diameter (Fig. 3A, Supplementary Fig. 1). In addition, Western Blot showed the positively expressed exosome markers CD63, CD81, and TSG101 in CAF-Exo and NF-Exo (Fig. 3B). To identify whether exosomes secreted by CAFs can be internalized by RCC cells, we pre-treated CAFs with DiIC_18_ and added these DiIC_18_ labeled exosomes into ACHN and 786-O culture medium. As expected, we could observe red fluorescence in ACHN and 786-O cells treated with DiIC_18_ labeled exosomes (Fig. 3C), suggesting the internalization of CAF exosomes by RCC cells. Whereafter, we carried out sphere formation, colony formation, and EdU assays to evaluate the effect of NF-Exo and CAF-Exo on RCC cells. As shown in Fig. 3D–F, CAF-Exo significantly enhanced the sphere formation, colony formation, and proliferation abilities of RCC cells compared with NF-Exo. Consistently, Western Blot and RT-qPCR also revealed the obviously increased protein and mRNA levels of OCT4 and ALDH1A1 in RCC cells treated with CAF-Exo rather than NF-Exo (Fig. 3G, H). These results proved that CAFs promoted the cancer stemness of RCC cells via delivering exosomes.

### CAFs deliver exosomal miR-181d-5p to RCC cells

**Fig. 4:**
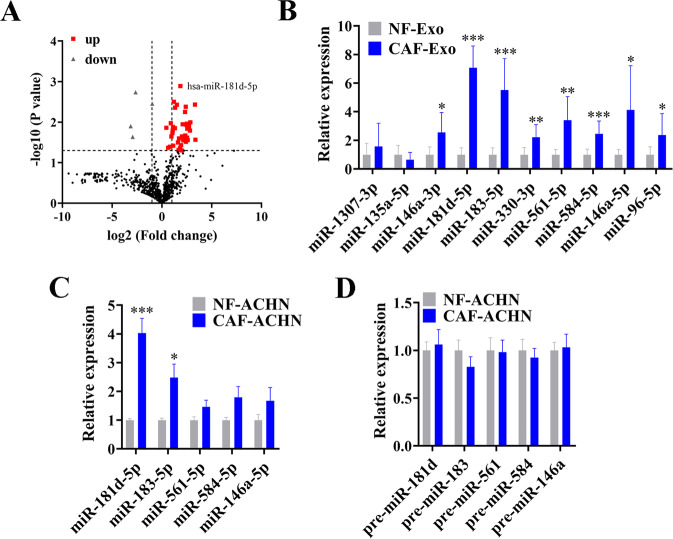
CAFs deliver exosomal miR-181d-5p to RCC cells. **A** Volcano Plot of differentially expressed miRNAs profile between CAF-Exo and NF-Exo by small RNA sequence, *n* = 3; **B** RT-qPCR analysis of exosomal miRNAs in NF-Exo and CAF-Exo, *n* = 8, normalized to U6; **C** RT-qPCR analysis of miRNAs expression in ACHN cells after treated with NF-Exo or CAF-Exo, normalized to U6; **D** RT-qPCR analysis of pre-miRNAs expression in ACHN cells after treated with NF-Exo or CAF-Exo, normalized to U6. Cell experiment was repeated three times independently, *P* value between the two groups was obtained by Unpaired *t* test. *P < 0.05; **P < 0.01; ***P < 0.001.

Seeing that miRNAs are important functional molecules in exosomes, we focused on the critical exosomal miRNAs transferred from CAFs to RCC cells. To confirm whether CAFs can secret exosomal miRNAs to RCC cells, we transfected CAFs with Cy3-tagged miRNA control, and then collected the exosomes in the culture medium to treat RCC cells. As expected, the fluorescently labeled miRNA control could be observed in RCC cells via confocal microscopy (Supplementary Fig. 2). Whereafter, we performed small RNA sequence to analyze the miRNA profile of CAF-Exo and NF-Exo (Fig. 4A), the detailed differentially expressed miRNAs list can be seen in Supplementary Table 2. Among the differentially expressed exosomal miRNAs, miR-1307-3p, miR-135a-5p, miR-146a-3p, miR-181d-5p, miR-183-5p, miR-330-3p, miR-561-5p, miR-584-5p, miR-146a-5p, and miR-96-5p were top 10 significantly upregulated miRNAs in CAF-Exo compared to NF-Exo, which were further analyzed by RT-qPCR (Fig. 4B). Based on these, we selected 5 distinctly upregulated miRNAs (miR-181d-5p, miR-183-5p, miR-561-5p, miR-584-5p, and miR-146a-5p), and detected the expression of these miRNAs and their corresponding pre-miRNAs in CAF-Exo or NF-Exo pre-treated ACHN cells. The results showed that miR-181d-5p and miR-183-5p were obviously enriched in RCC cells after treated with CAF-Exo, whereas their corresponding pre-miRNAs maintained invariant (Fig. 4C, D), suggesting that these mature miRNAs were directly transferred from CAFs to RCC cells via exosomes. We focused on the miR-181d-5p in the following experiments on account of its largest fold-change and unclear function in RCC progression.

### Exosomal miR-181d-5p secreted by CAFs enhance the cancer stemness of RCC cells

**Fig. 5:**
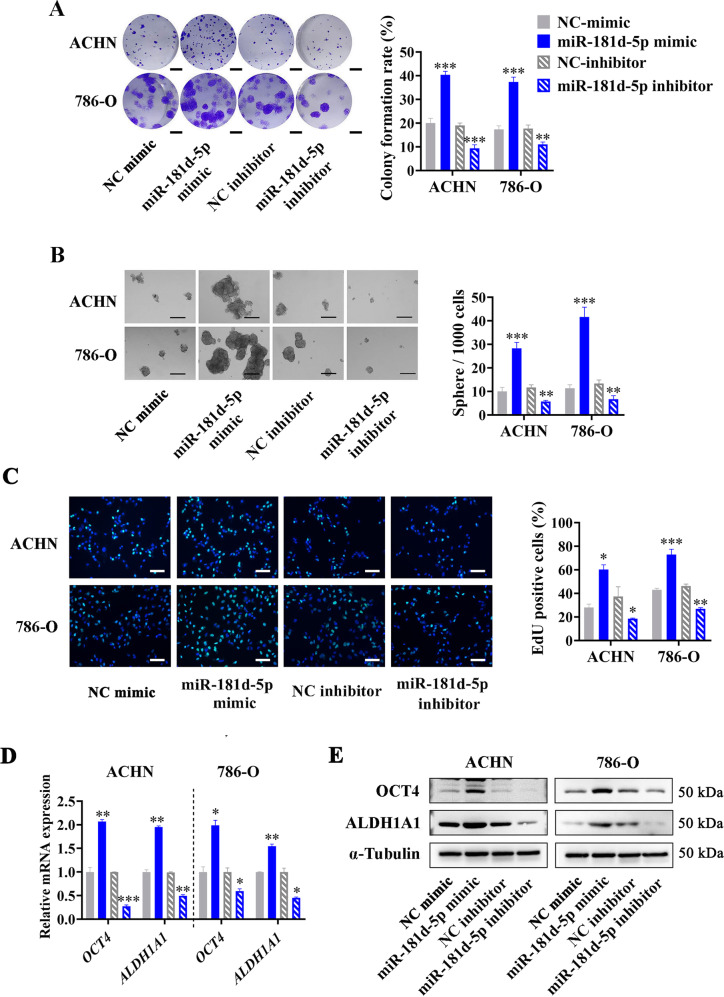
miR-181d-5p enhances the cancer stemness of RCC cells. **A** Representative images (Left) and histogram statistics (Right) of colony formation in ACHN and 786-O cells transfected with miR-181d-5p mimics, inhibitors, or corresponding controls, scale bar = 0.5 cm. **B** Representative images (Left) and histogram statistics (Right) of sphere formation in ACHN and 786-O cells transfected with miR-181d-5p mimics, inhibitors, or corresponding controls, scale bar = 100 µm. **C** Representative images (Left) and histogram statistics (Right) of EdU assay in ACHN and 786-O cells transfected with miR-181d-5p mimics, inhibitors, or corresponding controls, scale bar = 50 µm. **D** RT-qPCR analysis of mRNA levels in ACHN and 786-O cells transfected with miR-181d-5p mimics, inhibitors, or corresponding controls, normalized to *β-actin*. **E** Western blot analysis of protein levels in ACHN and 786-O cells transfected with miR-181d-5p mimics, inhibitors, or corresponding controls, normalized to α-Tubulin. Cell experiment was repeated three times independently, *P* value between two groups was obtained by Unpaired *t* test. *P < 0.05; **P < 0.01; ***P < 0.001.

To first explore the effect of miR-181d-5p on the cancer stemness of RCC cells, we artificially altered the miR-181d-5p expression level in ACHN and 786-O cell lines by transfecting miR-181d-5p mimics, inhibitors, and corresponding controls. Colony formation assay showed that upregulating miR-181d-5p expression level in RCC cells obviously increased the colony formation ability, while downregulating miR-181d-5p expression level significantly reduced the cell colony formation rate (Fig. 5A). Further sphere formation assay showed the increased cell sphere formation ability in miR-181d-5p mimic group compared to miR-NC mimic group, and reduced cell sphere formation in miR-181d-5p inhibitor group compared to miR-NC inhibitor group (Fig. 5B). The same results were also observed in the EdU assay, miR-181d-5p could obviously promote the proliferation ability of RCC cells (Fig. 5C). To analyze the expression of cancer stemness relative genes in RCC cells with dysregulated miR-181d-5p expression, RT-qPCR assays showed that the mRNA of *OCT4* and *ALDH1A1* were distinctly upregulated in miR-181d-5p mimic group and decreased in miR-181d-5p inhibitor group (Fig. 5D). Consistently, Western Blot also confirmed the increased protein levels of OCT4 and ALDH1A1 in miR-181d-5p mimics group and decreased protein levels in miR-181d-5p inhibitors group (Fig. 5E). These results demonstrated the important function of miR-181d-5p on prompting RCC stemness.

As miR-181d-5p has been reported to affect metastasis in breast cancer [22] and liver cancer [23], we also performed a cell scratch test and transwell assay in RCC cells, and found that upregulating miR-181d-5p expression level obviously increased the cell migration and invasion abilities, while downregulating miR-181d-5p showed the opposite effects (Supplementary Fig. 3A, B). We further detected the EMT markers in RCC cells transfected with miR-181d-5p mimics or inhibitors, and the results showed that overexpression miR-181d-5p could significantly upregulate the N-cadherin protein level and reduce the E-cadherin protein level, while blocking miR-181d-5p in RCC cells obviously decreased N-cadherin protein expression and enhanced the E-cadherin protein expression (Supplementary Fig. 3C). Hence, these results proved miR-181d-5p could also promote the migration, invasion, and EMT of RCC cells.

**Fig. 6:**
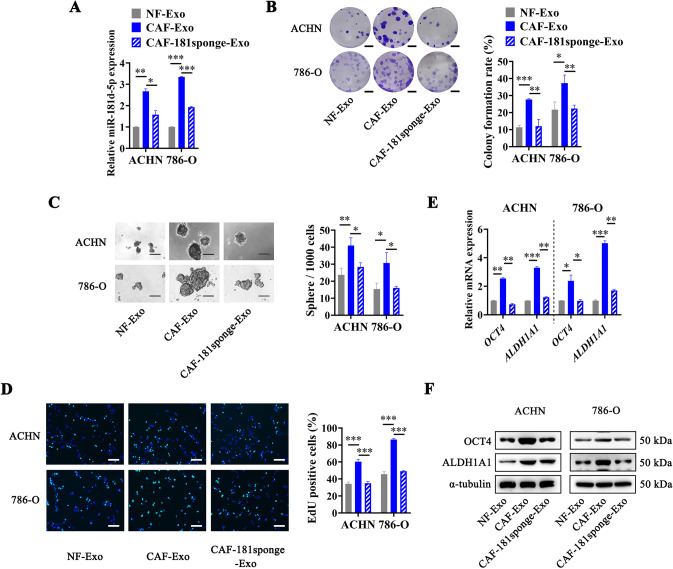
CAF-secreted exosomal miR-181d-5p enhances the cancer stemness of RCC cells. **A** RT-qPCR analysis of miR-181d-5p expression in RCC cells treated with NF-Exo, CAF-Exo, or CAF-181sponge-Exo, normalized to U6; **B** Representative images (Left) and histogram statistics (Right) of colony formation in RCC cells treated with NF-Exo, CAF-Exo, or CAF-181sponge-Exo, scale bar = 0.5 cm; **C** Representative images (Left) and histogram statistics (Right) of sphere formation in RCC cells treated with NF-Exo, CAF-Exo, or CAF-181sponge-Exo, scale bar = 100 µm; **D** Representative images (Left) and histogram statistics (Right) of EdU assay in RCC cells treated with NF-Exo, CAF-Exo, or CAF-181sponge-Exo, scale bar = 50 µm; **E** RT-qPCR analysis of mRNA levels in RCC cells treated with NF-Exo, CAF-Exo, or CAF-181sponge-Exo, normalized to *β-actin*; **F** Western Blot analysis of protein levels in RCC cells treated with NF-Exo, CAF-Exo, or CAF-181sponge-Exo, normalized to α-Tubulin. Cell experiment was repeated three times independently, *P* value between two groups was obtained by Unpaired *t* test. *P < 0.05; **P < 0.01; ***P < 0.001.

To further confirm the key role of exosomal miR-181d-5p from CAFs, we constructed miR-181d-5p specific sponge lentivirus to infect CAFs to reduce the levels of miR-181d-5p in CAFs-delivered exosomes (Fig. 6A). Then we treated RCC cells with NF-Exo, CAF-Exo, or CAF-181sponge-Exo, and found that bringing down the miR-181d-5p levels in CAFs-delivered exosomes could significantly inhibit the effect of CAFs-delivered exosomes on promoting the sphere formation, colony formation, and proliferation of RCC cells (Fig. 6B–D). Moreover, RT-qPCR and Western Blot assays showed that the expression levels of OCT4 and ALDH1A1 in RCC cells treated with CAF-181sponge-Exo were obviously decreased compared with that treated with CAF-Exo (Fig. 6E, F). The above results indicated that CAFs-delivered exosomal miR-181d-5p played a crucial role in enhancing the cancer stemness of RCC cells.

### RNF43 is a direct target of miR-181d-5p

**Fig. 7:**
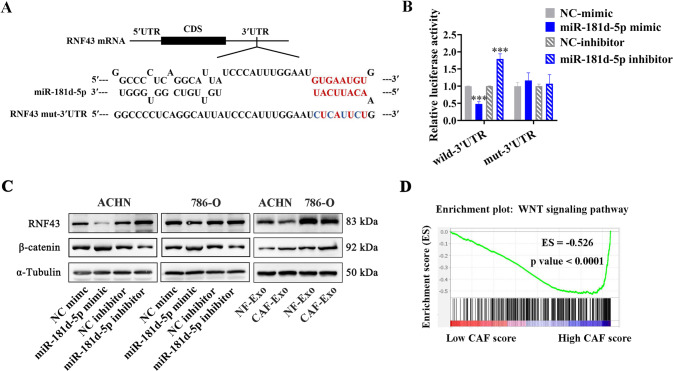
RNF43 is a direct target of miR-181d-5p. **A** predictive interaction of miR-181d-5p in *RNF43* mRNA 3′-UTR by TargetScan and RNAhybrid, and mutation of the indicated nucleotides in seed sequences. **B** Dual-luciferase reporter assays of the effects of miR-181d-5p mimics and inhibitors on wild-3′-UTR and mut-3′-UTR in ACHN cells. **C** Western Blot analysis of RNF43 and β-catenin levels in ACHN and 786-O cells transfected with miR-181d-5p mimics, inhibitors, or corresponding controls, as well as in ACHN and 786-O cells pre-treated with NF-Exo or CAF-Exo. **D** Enrichment profile of the WNT signaling pathway in high vs low CAF score RCC tumors using GSEA, (ES = -0.526, *P* < 0.0001). Cell experiment was repeated three times independently, *P* value between two groups was obtained by Unpaired *t* test. ***P < 0.001.

To ascertain the downstream targets of miR-181d-5p regulation, we performed bioinformatic prediction by two commonly used algorithm, Targetscan [24] and RNAhybrid [25]. Ring finger protein 43 (RNF43) was screened as a potential target of miR-181d-5p. The complementarity sequence of miR-181d-5p to the 3′-UTR of *RNF43* mRNA was shown in Fig. 7A. To substantiate that miR-181d-5p regulates RNF43 protein level via the presumed binding sites in the 3′-UTR of *RNF43* mRNA, we constructed a dual-luciferase reporter plasmid, with the 200 bp fragment of *RNF43* 3′-UTR containing the predicated binding sites inserted downstream of the firefly luciferase gene. Meanwhile, the plasmid containing mutated miRNA seed sequence complementary sites was also constructed (Fig. 7A). As a result, overexpressing miR-181d-5p significantly decreased the luciferase activity of wild-3′-UTR plasmid, and inversely, blocking miR-181d-5p obviously enhanced the luciferase activity. However, the luciferase activity of mut-3′-UTR plasmid was not changed (Fig. 7B).

RNF43 is an E3 ubiquitin ligase, and has been reported as a pivotal negative regulator of the Wnt/β-catenin signal activity through triggering Frizzled family degradation via ubiquitination, possessing important significance in inactivating Wnt/β-catenin signaling and CSCs-targeted therapy [26, 27]. To investigate the effects of miR-181d-5p on RNF43 level and Wnt/β-catenin signal activity, we performed western blot assay and found that RNF43 protein expression was notably suppressed when upregulating miR-181d-5p levels, and increased when blocking the miR-181d-5p expression. Inversely, the protein level of β-catenin was increased when upregulating miR-181d-5p levels, and decreased when blocking the miR-181d-5p expression (Fig. 7C). Moreover, RNF43 protein level was obviously reduced in RCC cells treated with CAF-Exo compared to NF-Exo, and β-catenin protein levels changed oppositely (Fig. 7C). Our results indicated that CAFs-delivered exosomal miR-181d-5p might promote RCC stemness via directly targeting RNF43 and thus activating Wnt/β-catenin signaling in RCC cells.

The Wnt/β-catenin signaling pathway is considered as the crucial pathway to regulate the cancer stemness of RCC [28]. To study the effects of the enrichment of CAFs on Wnt/β-catenin signal activity in clinical RCC tissues, we generated a CAF-specific gene signature as previously reported [29] to perform Gene set enrichment analysis (GSEA) using data from TCGA database. The GSEA results showed a significant enrichment for the Wnt/β-catenin signaling pathway gene set when compared high CAF-scored tumors versus low CAF-scored tumors (Fig. 7D), indicating a close relationship between CAFs and Wnt/β-catenin signaling activity in RCC.

**Fig. 8:**
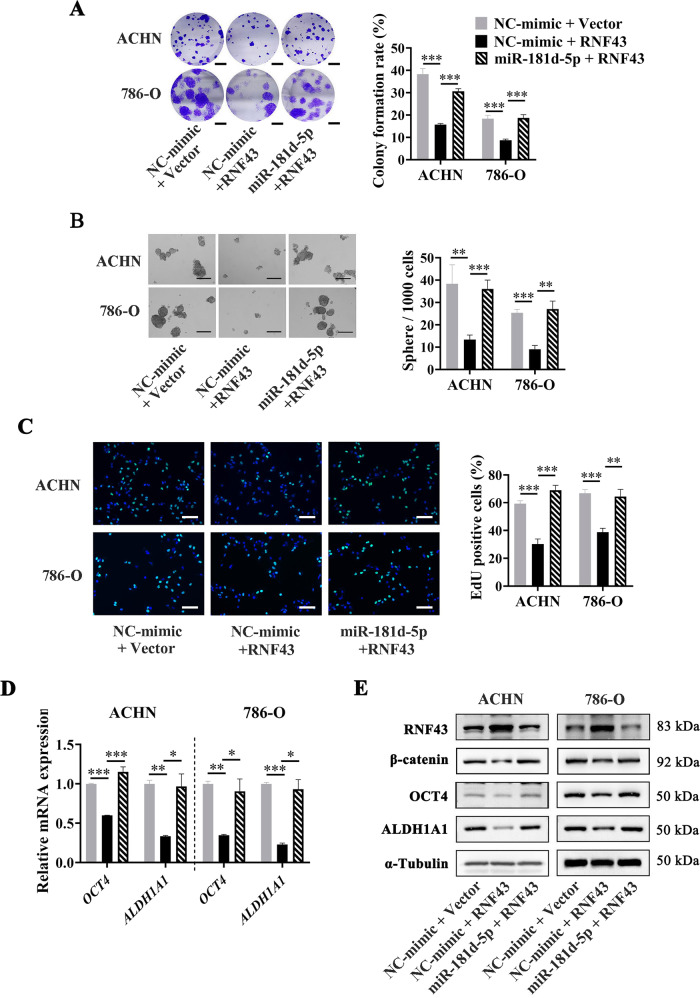
miR-181d-5p promotes cancer stemness and RCC progression by suppressing RNF43 and activating Wnt/β-catenin signal in vitro. **A** Representative images (Left) and histogram statistics (Right) of colony formation in RCC cells transfected with RNF43, or RNF43 plus miR-181d-5p mimics, scale bar = 0.5 cm. **B** Representative images (Left) and histogram statistics (Right) of sphere formation in RCC cells transfected with RNF43, or RNF43 plus miR-181d-5p mimics, scale bar = 100 µm. **C** Representative images (Left) and histogram statistics (Right) of EdU assay in RCC cells transfected with RNF43, or RNF43 plus miR-181d-5p mimics, scale bar = 50 µm. **D** RT-qPCR analysis of mRNA levels in RCC cells transfected with RNF43, or RNF43 plus miR-181d-5p mimics, normalized to *β-actin*; **E** Western Blot analysis of protein levels in RCC cells transfected with RNF43, or RNF43 plus miR-181d-5p mimics, normalized to α-Tubulin. Cell experiment was repeated three times independently, *P* value between two groups was obtained by Unpaired *t* test. *P < 0.05; **P < 0.01; ***P < 0.001.

To further explore the role of miR-181d-5p/RNF43/Wnt signaling pathway axis in RCC stemness, we constructed RNF43 vector with the 200 bp fragment of its 3′-UTR containing the aforesaid miR-181d-5p binding sites. Colony formation, sphere formation and EdU assays were performed in RCC cells transfected with RNF43 vector or both RNF43 vector and miR-181d-5p mimics. The result showed that overexpression of RNF43 obviously attenuated the abilities of cell colony formation, sphere formation, and proliferation, while simultaneously transfecting with miR-181d-5p mimics could restore these abilities (Fig. 8A–C). After that, we detected the mRNA levels of *OCT4* and *ALDH1A1*, cancer stemness markers of RCC cells that were also proved to be regulated by Wnt/β-catenin signal activity [30]. As shown in Fig. 8D, the *OCT4* and *ALDH1A1* expression levels were significantly downregulated in cells overexpressing RNF43, and reverted when simultaneously transfecting with miR-181d-5p mimics (Fig. 8D). We further detected the protein levels of RNF43, β-catenin, OCT4, and ALDH1A1, and found that the protein levels of β-catenin, OCT4, and ALDH1A1 were decreased when overexpressing RNF43, however, these effects could be reversed by simultaneously transfecting with miR-181d-5p mimics (Fig. 8E). In summary, these results revealed that CAFs-secreted exosomal miR-181d-5p directly suppressed the RNF43 protein expression in RCC cells, and thus activated Wnt/β-catenin signal and promoted RCC stemness.

### miR-181d-5p promotes cancer stemness and RCC progression by targeting RNF43 and activating Wnt/β-catenin signal in vivo

**Fig. 9:**
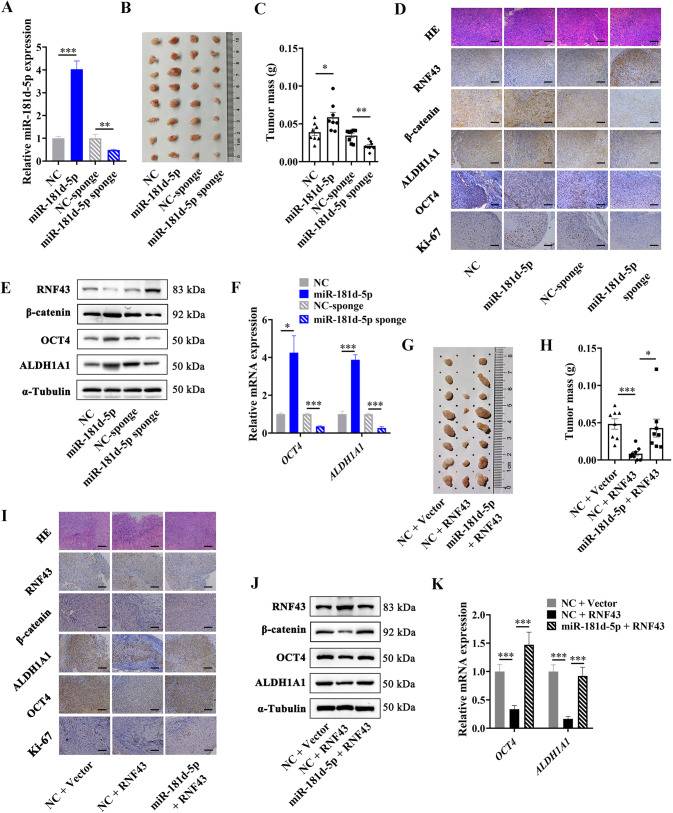
miR-181d-5p promotes cancer stemness and RCC progression by suppressing RNF43 and activating Wnt/β-catenin signal in vivo. **A** RT-qPCR analysis of miR-181d-5p levels in ACHN cells stably infected with miR-181d-5p overexpressing lentivirus or miR-181d-5p sponge lentivirus. **B** Representative images of tumors from mice of each group; **C** Quantitative analysis of the tumor weights in **B**. **D** Representative images of H&E staining and IHC staining for RNF43, β-catenin, ALDH1A1, OCT4, and Ki67 of tumors from the implanted mice, scale bar = 100 µm. **E** Western Blot analysis of RNF43, β-catenin, ALDH1A1, and OCT4 protein levels in tumors from the implanted mice, normalized to α-Tubulin; **F** RT-qPCR analysis of *ALDH1A1* and *OCT4* mRNA levels in tumors from the implanted mice, normalized to *β-actin*. **G** Representative images of tumors from mice of each group. **H** Quantitative analysis of the tumor weights in **G**. **I** Representative images of H&E staining and IHC staining for RNF43, β-catenin, ALDH1A1, OCT4, and Ki67 of tumors from the implanted mice, scale bar = 100 µm. **J** Western blot analysis of RNF43, β-catenin, ALDH1A1, and OCT4 protein levels in tumors from the implanted mice, normalized to α-tubulin. **K** RT-qPCR analysis of *ALDH1A1* and *OCT4* mRNA levels in tumors from the implanted mice, normalized to *β-actin*. *P* value between the two groups was obtained by Unpaired *t* test. *P < 0.05; **P < 0.01; ***P < 0.001.

To probe into the effects of miR-181d-5p/RNF43/Wnt signaling pathway axis on cancer stemness and RCC progression in vivo, we firstly constructed miR-181d-5p stably overexpressed or blocked ACHN cells with miR-181d-5p overexpression lentivirus or sponge lentivirus, respectively (Fig. 9A). The cells in each group were implanted into the armpits of nude mice subcutaneously, then the tumor growth was evaluated for 30 days. As shown in Fig. 9B, C, tumor size and mass were obviously accelerated in miR-181d-5p overexpressed group, and reduced when miR-181d-5p was blocked. Western Blot and IHC staining assays of tumors showed that RNF43 protein levels decreased in miR-181d-5p overexpressed group, and rose when miR-181d-5p was blocked (Fig. 9D, E). Whereas Ki67, β-catenin, OCT4, and ALDH1A1 were obviously increased following miR-181d-5p upregulated, and decreased in miR-181d-5p sponge group, consistently with in vitro experiments (Fig. 9D–F). We also investigated the N-cadherin and E-cadherin levels in the tumors of each group, and found N-cadherin was significantly enriched in miR-181d-5p overexpressed group, and decreased when miR-181d-5p was blocked, whereas E-cadherin was the opposite change. These results proved the EMT promotion effect of miR-181d-5p in vivo (Supplementary Fig. 3D, E).

Moreover, a rescue experiment in vivo was carried out. ACHN cells were stably infected with RNF43 overexpressing lentivirus, or co-overexpressed RNF43 and miR-181d-5p lentivirus. Subcutaneous xenografted results showed that overexpressing RNF43 in RCC cells dramatically reduced the tumor size and mass when compared to the control group, while co-overexpressing RNF43 with miR-181d-5p could block the effects of RNF43 (Fig. 9G, H). In according with in vitro study, Ki67, β-catenin, OCT4, and ALDH1A1 levels greatly downregulated in RNF43 overexpressed tumors, and restored when co-overexpressing RNF43 with miR-181d-5p (Fig. 9I–K). Above all, these results proved that miR-181d-5p promotes cancer stemness and RCC progression by targeting RNF43 and activating Wnt/β-catenin signal in vivo.

## Discussion

The CSCs model declares that tumors are highly dependent on a subset of cancer cells possessing self-renewal abilities and multilineage differentiation potential in tumorigenesis, metastasis, chemoresistance, and recurrence [31, 32]. Recent studies report that cancer stemness is not a fixed but flexible ability of cancer cells that can be acquired or lost [12]. There have been found that multiple factors can regulate the maintenance of the stemness of cancer cells [33, 34]. Among which, CAFs, as the major component of tumor microenvironment, are reported to tightly regulate cancer stemness in several types of cancer [35]. In breast cancer, CD10^+^GPR77^+^ CAFs are found to sustain cancer stemness through secreting IL-6 and IL-8, thus mediating tumorigenesis and therapy resistance [8]. In colorectal cancer, CAFs can control cancer cell plasticity by Netrin-1 [13]. In pancreatic cancer, CAFs can secret OPN/SPP1 to regulate the CD44 axis in cancer cells enhancing cancer stemness [36]. However, in RCC, the function and molecule mechanism of CAFs in cancer stemness maintenance remain unclear. In this study, we revealed that the enrichment of CAFs was positively correlated with tumor progression and cancer stemness in RCC, furthermore, exosomal miR-181d-5p secreted by CAFs played a key role in promoting cancer stemness and RCC progression by targeting RNF43.

Exosomal miRNAs are powerful communication factors in tumor microenvironment, which can be delivered by multiple kinds of cell types, and then enriched and functioned in recipient cells [15]. Such as, exosomal miR-9 and miR-181a derived from breast cancer cells activate the JAK/STAT signaling pathway in eMDSCs, thus promoting the expansion of eMDSCs [37]. CAFs secreted exosomal miR-196a to cancer cells, targeting CDKN1B and ING5 and mediating cisplatin resistance in head and neck cancer [38]. Here, we performed small RNA sequences to explore the key miRNAs in CAF exosomes of RCC, and ultimately screened miR-181d-5p significantly enriched in CAF exosomes and RCC cells pre-treated by CAF-Exo. Studies regarding to the role of miR-181d-5p in tumors are deficient and controversial. Several studies reported that miR-181d-5p restrained cell proliferation and metastasis in osteosarcoma and non-small-cell lung cancer [39, 40]. Whereas, a recent study demonstrated that hepatoma cell-derived exosomal miR-181d-5p could facilitate liver cancer metastasis through FAK/Src pathway [22]. Besides, in breast cancer, CAFs exosomal miR-181d-5p facilitated EMT by regulating CDX2/HOXA5 [21]. However, the function of miR-181d-5p in RCC has not been reported. In our study, we demonstrated that miR-181d-5p played an oncogenic role by suppressing RNF43 and activating Wnt/β-catenin signal in RCC, thus promoting cancer stemness and tumor progression.

RNF43 is an E3 ubiquitin ligase, it not only negatively modulates Wnt/β-catenin signaling through triggering Frizzled family degradation via ubiquitination, but also is regulated by Wnt/β-catenin signaling itself [26, 27]. On account of its crucial function in restraining the Wnt/β-catenin signal activity, RNF43 is widely recognized as a tumor suppressor in various cancers, such as gastric cancer, colorectal cancer, and pancreatic carcinoma [26, 41, 42]. Up to now, Wnt/β-catenin signaling has been considered as the key factor in regulating cancer stem cells, and masses of cancer stemness-related genes (*OCT4*, *ALDH1A1*, *LGR5*, *CD44*, *c-Myc*, *SOX2,* and so on) are regulated by Wnt/β-catenin signaling [43, 44]. Thus, targeting Wnt/β-catenin signaling might prove to be an effective therapeutic strategy for stemness restraint and cancer treatment. Currently, numerous pharmacological antagonists of the Wnt pathway have been investigated, and part of these agents have reached clinical testing [34]. RNF43, as the pivotal negative regulator of the Wnt signaling, possesses important significance in inactivating Wnt signaling and CSCs-targeted therapy [27]. In the present study, we demonstrated CAFs-secreted miR-181d-5p directly suppressed RNF43 protein expression in RCC cells, suggesting a novel CAFs exosomal miRNAs-based strategy for RCC treatment.

**Fig. 10:**
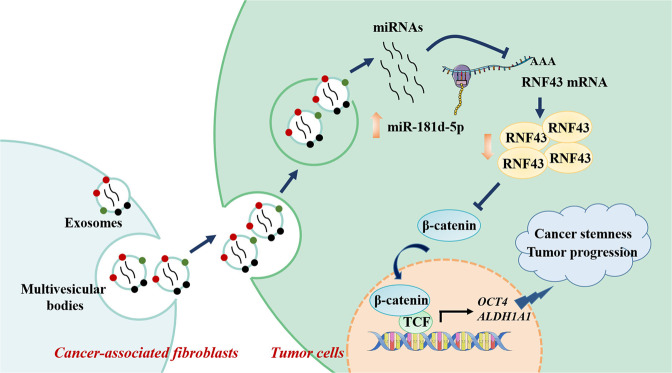
A schematic diagram of the mechanism. CAFs secreted exosomal miR-181d-5p to RCC cells, suppressed the expression of RNF43, activated Wnt/β-catenin signaling pathway, thus promoted cancer stemness and tumor progression.

As summarized in our proposed scheme model in Fig. 10, our data revealed that the enrichment of CAFs was positively associated with cancer stemness and RCC progression, moreover, CAFs-delivered exosomal miR-181d-5p directly targeted RNF43, activated the Wnt/β-catenin signaling, thus enhanced cancer stemness and mediated RCC progression. These results unlocked a novel promising CAFs exosomal miRNAs-based strategy for RCC treatment.

## Materials and methods

### Specimens, IHC staining, and H&E staining

All the clinical samples were obtained from the Nanjing Drum Tower Hospital (Nanjing, China). More specifically, the fresh tissues for CAFs and NFs isolation, as well as protein or RNA extraction, were obtained from patients undergoing nephrectomy for renal tumor immediately, and the tissue microarray (TMA) chips containing a total of 141 RCC samples for immunohistochemistry (IHC) staining were constructed by paraffin-embedded samples obtained from patients received surgical resection from 2010 to 2015. The tissues collection and analysis were conducted in keeping with the guidelines of the Declaration of Helsinki and also approved by the Ethics Committee of Nanjing Drum Tower Hospital. Pathological differentiation and clinical stage were confirmed referring to the World Health Organization (WHO) classification.

IHC staining and hematoxylin and eosin (H&E) staining were performed in accordance with standard protocols by Cell Signaling Technology, the antibodies were as follows: anti-α-SMA (abcam, Cambridge, United Kingdom, ab5694), anti-CD105 (proteintech, Wuhan, China,10862-1-AP), anti-RNF43 antibody (bioss, Beijing, China, bs-24331R), anti-β-catenin antibody (proteintech, 51067-2-AP), anti-ALDH1A1 antibody (proteintech, 15910-1-AP), anti-OCT4 antibody (proteintech, 11263-1-AP), anti-N-cadherin antibody (proteintech, 66219-1-Ig), and anti-E-cadherin antibody (proteintech, 20874-1-AP). An established immunoreactivity scoring (IRS) system was applied to semi-quantitatively score the expression of αSMA and CD105. Specifically, IRS is the product of the proportion score (0-4) and the staining intensity score (0–3), ranging from 0 to12. Two blinded pathologists were enrolled to grade the slides, and the mean score was calculated as the exact IRS score (Supplementary Table 1).

### Cell culture and co-culture

We purchased the human renal carcinoma cell lines ACHN and 786-O from the Shanghai Institute of Cell Biology (Shanghai, China). All the cell lines were identified by short tandem repeat (STR) analysis. ACHN and 786-O were cultured with Gibco DMEM and 1640 medium (Gibco, Carlsbad, United States, 11965118, 72400047), respectively. CAFs and NFs were isolated from fresh RCC tissues and their paired normal kidney tissues respectively. Specifically, tissues were cut into small pieces and then digested by collagenase type I (1.5 mg/mL, SCR103) and hyaluronidase (125 U/mL, H3506) (Sigma-Aldrich, Darmstadt, Germany) in 10% FBS DMEM culture medium at 37 °C with agitation for 1 h. Thereafter, to isolate primary fibroblasts, the digested tissue pieces were seeded in dishes and fostered in 10% FBS DMEM culture medium for 2–3 days at 37 °C in the presence of 5% CO_2_. After the suspension cells and tissue debris were removed, the majority of adherent cells were fibroblasts. Then the cells were further incubated for 3–5 days to acquire purer fibroblasts, meanwhile, fresh medium was replaced every day. We used the third to eighth passages of the primary fibroblasts in our study.

For co‑culture assay, a 0.4 µm transwell chamber was used. 5 × 10^4^ ACHN or 786 cells were cultured in the lower compartment and 1 × 10^4^ CAFs or NFs were seed in the upper chamber, the co-culture system was supplemented with 10% exosome‑free FBS DMEM medium, and cultured at 37˚C. CAFs were treated with or without 10 μM GW4869 (Sigma-Aldrich, D1692) for 24 h before seeding. RCC cells were collected for further assays after 48 h of co-culture.

### Extraction and identification of exosomes

To isolate exosomes from the cell culture medium, cells were seeded in dishes and cultured in 10% exosome-free FBS DMEM medium for 48 h, then the cell culture medium was collected for exosomes isolation using Total Exosome Isolation Kit (Invitrogen, California, United States, 4478359) following the manufacturer’s instructions [45]. GW4869 was used to inhibit exosome release at 10 µM. Western blotting was used to identify the exosome markers CD63, CD81, and TSG101. Transmission electron microscopy was applied to observe the morphology characteristics, and Nanoparticle tracking analysis (NanoSight™) was used to analyze the size distribution profile of isolated exosomes [45]. To investigate the effects of CAFs-delivered or NFs-delivered exosomes on RCC cells, 20 ng/ml individual exosomes were used to treat RCC cells for 48 h before further assays.

### Cell transfection

MiR-181d-5p mimics and inhibitors were constructed from RiboBio (Guangzhou, China). The RNF43 vector contained a 200 bp fragment of its 3′-UTR with miR-181d-5p binding sites, and the control empty plasmid (pcDNA3.1) were constructed and purchased from YouBio (Changsha, China). Transfection was carried out with Lipofectamine 3000 reagents (Invitrogen).

### RNA extraction and qRT-PCR

TRIzol reagent (Invitrogen, 15596018) was used to extract total RNA from cells and exosomes. Reverse transcription was conducted by miRNA 1st Strand cDNA Synthesis Kit (Vazyme, Nanjing, China, MR101-01/02) for miRNA, and PrimeScript™ RT Master Mix (TaKaRa, Shiga, Japan, RR036A) for general genes, real-time PCR was carried out with ChamQ SYBR qPCR Master Mix (Vazyme, Q311-02/03) and performed on Applied Biosystems QuantStudio^TM^ 6 Flex Real-Time RCR System (Applied Biosystems, California, United States). The primer sequences were listed in Supplementary Table 3. U6 and *β-actin* were used as endogenous control to normalize miRNA and mRNA expression respectively. All reactions were performed in triplicate.

### Western blotting

Protein extraction and western blotting were performed as described previously [46]. The antibodies were as follows: anti-α-SMA (abcam, ab5694), Vimentin (Proteintech, 10366-1-AP), anti-E-cadherin antibody (proteintech, 20874-1-AP), anti-CD63 antibody (santa cruze, sc-5275), anti-CD81 antibody (proteintech, 27855-1-AP), anti-TSG101 antibody (Santa Cruz, California, United States, sc-7964), anti-RNF43 antibody (bioss, bs-24331R), anti-β-catenin antibody (proteintech, 51067-2-AP), anti-ALDH1A1 antibody (proteintech, 15910-1-AP), anti-OCT4 antibody (proteintech, 11263-1-AP), anti-N-cadherin antibody (proteintech, 66219-1-Ig), and anti-α-tubulin (abcam, ab7291). Protein levels were normalized to α-tubulin and analyzed by Image J software.

### Sphere formation assay

RCC cells were seeded (1250 cells/well) in ultra-low adhesion 24-well plates (Corning), and cultured in DMEM-F12 medium supplemented with 2% B27 (Invitrogen, 17504044), 10 ng/ml bFGF (Invitrogen, RP-8628), and 20 ng/mL EGF (Invitrogen, PHG0311L). The cells were cultured at 37 °C in the presence of 5% CO_2_ for 10 days, and then cell sphere formation was observed and mammospheres with a diameter >75 mm were counted.

### Colony formation assay

RCC cells were seeded (50 cells/well) in 12-well plates and then cultured at 37 °C for 1–2 weeks. For staining, cells were firstly fixed with pure methanol for 10–15 min, and then stained with crystal violet for another 10–15 min. We counted clones containing more than 50 cells under a microscope. The percentage of colonies formed was calculated as colony formation rate.

### EdU assay

RCC cells were seeded (2 × 10^4^ cells/well) in 48-well plates and then cultured at 37 °C for 12 h. EdU assay was performed using BeyoClick™ EdU Cell Proliferation Kit with Alexa Fluor 488 (Beyotime, Shanghai, China, C0071S) according to the manufacturer’s manual. The images were observed with a laser scanning confocal microscopy and analyzed by ImageJ software.

### Cell scratch test

RCC cells were seeded in 6-well plates, an artificial wound was created 24 h after transfection using a 200 μL pipette tip. Next, cells were cultured with 2% FBS culture medium for 20 h, and the images were taken at 0 and 20 h after the wound was created. The migration ratio was analyzed as following: the migration ratio = 1−the wound average width at 20 h/ the wound average width at 0 h.

### Transwell assay

8 µm pore transwell chambers (Corning, NY, USA) coated with matrigel (200 ng/mL; BD Biosciences, NY, USA, 354230) were used to analyse the invasive ability of cells according to the manufacturer’s protocol. After 20 h of incubation, cells invading into the lower surface of the membrane insert were fixed in 4% paraformaldehyde, and stained with 0.5% crystal violet. Five random fields were selected to count the number of cells passing through the chamber.

### Exosome tracing

CAFs were pre-treated by DiIC_18_ (Beyotime, C1036) or transfected with Cy3-labeled miRNA. After 24 h, exosomes in cell culture medium were isolated as described above. The isolated exosomes were incubated with recipient RCC cells for 12 h, and then observed by a confocal laser scanning microscopy.

### Immunofluorescence

Cells were firstly fixed using 4% paraformaldehyde, washed twice with PBS, and permeabilized with 0.2% TritonX-100. After blocking, incubating cells with primary antibodies specific for α-SMA (Abcam, ab5694), Vimentin (Proteintech, 10366-1-AP), or E-cadherin antibody (Proteintech, 20874-1-AP), overnight at 4 °C. After washing, cells were incubated with fluor-conjugated secondary antibodies (Invitrogen, A-11001) for 1 h, and DAPI for 5 min at room temperature. We observed the results with a laser scanning confocal microscopy.

### Small RNA sequencing for exosomal miRNAs

Exosomes were isolated from three paired primary NFs and CAFs, respectively. Total RNA of exosomes was extracted as described above. After checking the quantity and integrity, 1.5 µg of total RNA for each sample was used to generate the small RNA library, and then the libraries were sequenced on an Illumina Hiseq 2500/2000 platform and further subjected to the following analyses. These small RNA libraries construction and preliminary bioinformatics analysis were performed by Lc-bio Technologies (Hangzhou, China). The raw sequencing data from this study have been deposited in Gene Expression Omnibus with the accession number: GSE213453.

### Luciferase reporter assay

To identify the direct binding of miR-181d-5p to RNF43, we transfected RCC cells with pMir-GLO dual-luciferase reporter vectors, which contained a 200 bp fragment of the RNF43 3′-UTR with predicted miR-181d-5p binding sites or mutant sites, and then increased or reduced miR-181d-5p expression level by co-transfecting with miRNA mimics or inhibitors. The pMir-GLO dual-luciferase reporter vector was constructed by TSINGKE (Nanjing, China). 24–48 h after transfection, we measured firefly and renilla activities with a dual-luciferase assay kit (Vazyme, DL101-01) according to the manufacturer’s manual.

### Bioinformatics analysis

We downloaded the RCC data from The Cancer Genome Atlas (TCGA), and used CAF score signature from Liu et al. [27]. Briefly, Liu et al. identified the seven key genes of the CAF score signature using Weighted Gene Co-expression Network Analysis, and in this study, we used the CAF score signature to distinguish the CAF-High group and the CAF-low group in RCC data from TCGA, and then performed GSEA to analyze CAF related pathways according to the manufacturer’s protocol.

### Xenograft in vivo models

Six-week-old male nude mice were purchased from the Model Animal Research Center of Nanjing University (Nanjing, China) and fed under specific pathogen-free conditions at Nanjing Drum Tower Hospital (Nanjing, China). Prior to the experiment, mice were randomly assigned to each group. Lentivirus vectors including miR-181d-5p, miR-181d-5p sponge, RNF43 vector containing a 200 bp fragment of its 3′-UTR with miR-181d-5p binding sites, and the corresponding controls were constructed and generated by Hesheng Bio (Shanghai, China), puromycin was used to select the lentivirus transfected ACHN cells. For xenograft subcutaneous implantations, 2 × 10^6^ ACHN cells were seeded into oxter of nude mice (8 mice/group). The sample size was chosen with adequate power on the basis of the literature and our previous experience [46]. After 30 days, mice were euthanized, and then the tumors were collected and analyzed. All animal care and handling procedures were performed in keeping with the NIH’s Guide for the Care and Use of Laboratory Animals and were approved by the Institutional Review Board of Nanjing Drum Tower Hospital (Nanjing, China). All investigators were blind to the treatment condition until after the completion of all data analyses.

### Statistical analysis

All experiments were repeated three times. Graph Pad Prism 8.0 software was used to perform the statistical analyses in this study. Student’s *t* test was performed to analyze the significance between the two groups. Quantitative values were selectively shown as the mean ± SD or mean ± SEM. Pearson’s correlation analysis was used to measure the linear relationship between the IHC score of α-SMA and CD105. Western Blot was analyzed with Image J software. *P* < 0.05 was identified statistically significant.

## Supplementary information

Supplementary Figures

Supplementary Table 1

Supplementary Table 2

Supplementary Table 3

Original data file

## Data Availability

The datasets supporting the conclusions of this article are included within the article and the additional file. More supporting data are available under reasonable request.
