# Supplementary material for: Cancer-associated fibroblasts promote the stemness and progression of renal cell carcinoma via exosomal miR-181d-5p
Source: Cell Death Discov. 2022 Nov 1;8:439. doi: 10.1038/s41420-022-01219-7 (PMC9626570; doi:10.1038/s41420-022-01219-7)
Supplement: Supplementary file 1 — Supplementary Figures [file 41420_2022_1219_MOESM1_ESM.docx]

**Cancer-associated fibroblasts promote the stemness and progression of renal cell carcinoma via** **exosomal miR-181d-5p**

Meng Ding^1#^, Xiaozhi Zhao^1#^, Xiaoqing Chen^1#^, Wenli Diao^1^, Yansheng Kan^2^, Wenmin Cao^1^, Wei Chen^1^, Bo Jiang^1^, Haixiang Qin^1^, Jie Gao^1^, Junlong Zhuang^1^, Qing Zhang^1^*, and Hongqian Guo^1^*

**Supplementary Materials**

Supplementary Figure 1

Supplementary Figure 2

Supplementary Figure 3


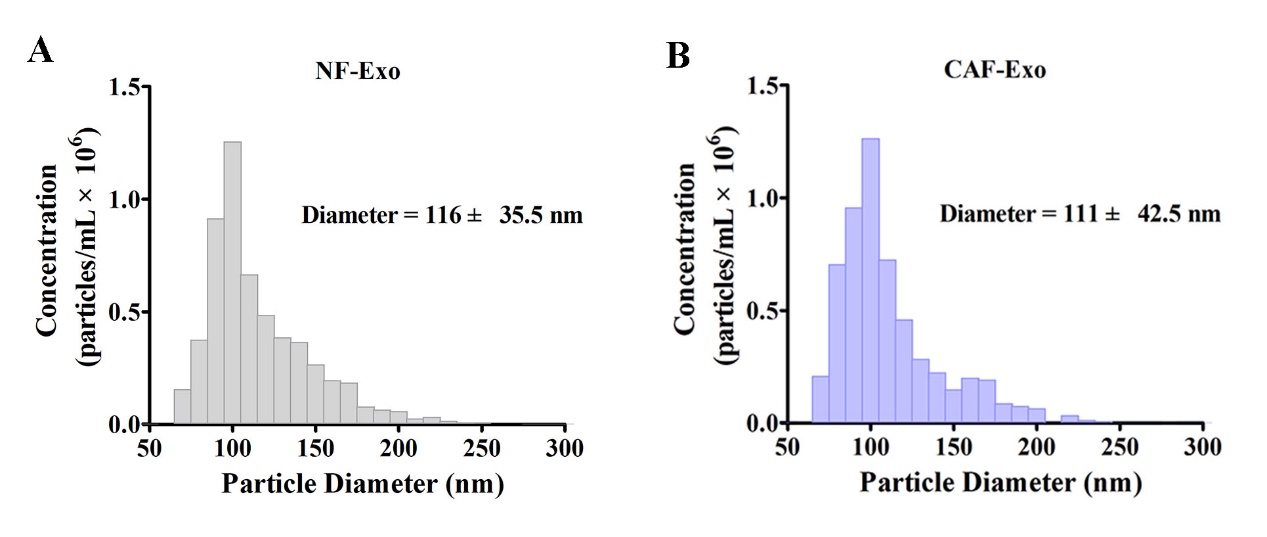


**Supplementary Figure 1**. NanoSight analysis of the isolated exosomes in CAFs and NFs.


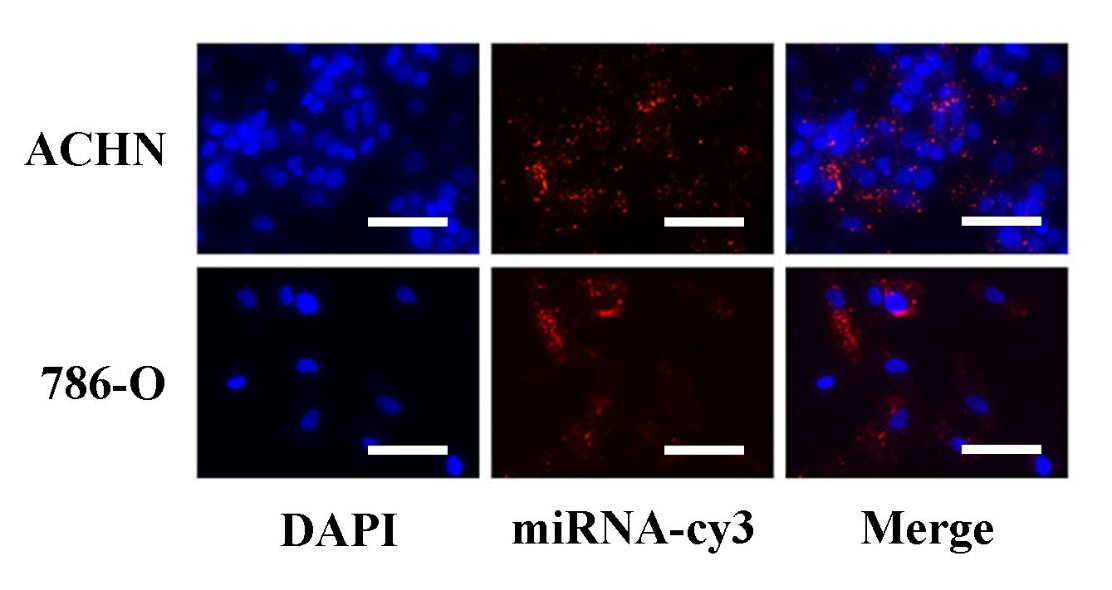


**Supplementary Figure 2**. Transfer of exosomes from CAFs to RCC cells examined by confocal microscope, scale bar = 50 µm.


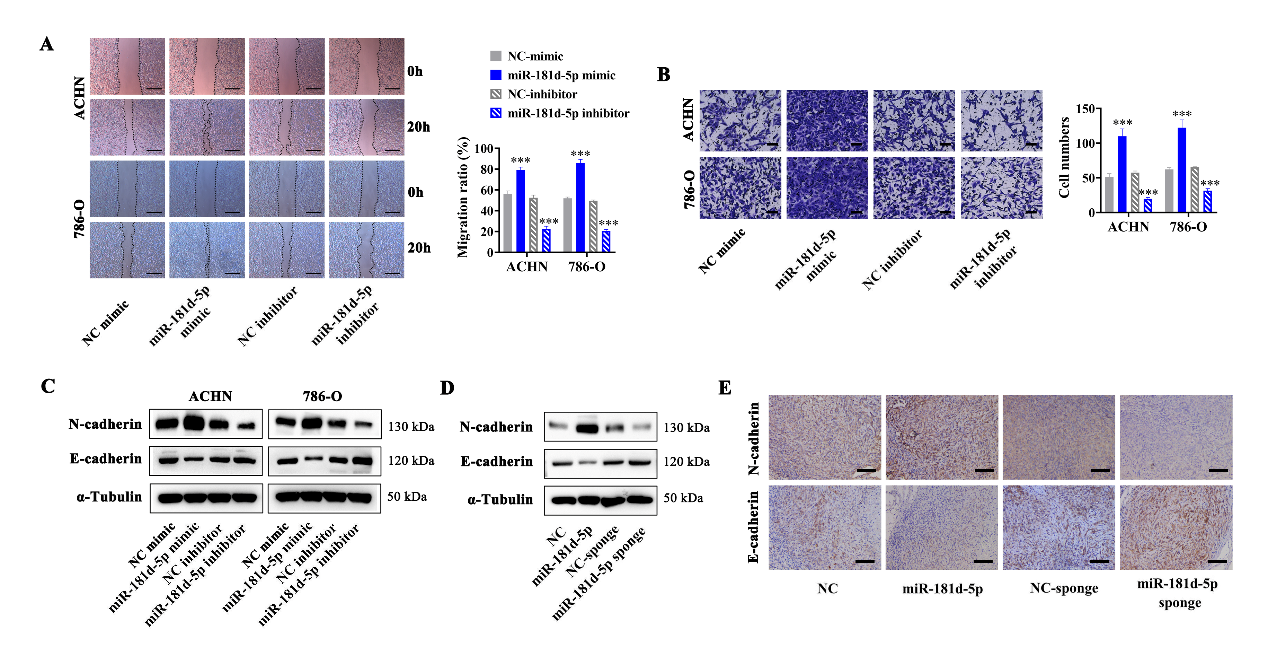


**Supplementary Figure 3. miR-181d-5p promote the migration and invasion of RCC cells *in vitro* and *in vivo*.** **A** Representative images (Left) and histogram statistics (Right) of Cell Scratch Test in ACHN and 786-O cells transfected with miR-181d-5p mimics, inhibitors, or corresponding controls, scale bar = 10 mm; **B** Representative images (Left) and histogram statistics (Right) of Transwell assay in ACHN and 786-O cells transfected with miR-181d-5p mimics, inhibitors, or corresponding controls, scale bar = 100 µm; **C** Western Blot analysis of protein levels in ACHN and 786-O cells transfected with miR-181d-5p mimics, inhibitors, or corresponding controls, normalized to α-Tubulin. **D** Western Blot analysis of N-cadherin and E-cadherin protein levels in tumors from the implanted mice, normalized to α-Tubulin; **E** Representative images of IHC staining for N-cadherin and E-cadherin of tumors from the implanted mice, scale bar = 100 µm. Cell experiment was repeated three times independently, *P* value between two groups was obtained by Unpaired t-test. *** *P* < 0.001.
