# Supplementary material for: Cancer-associated fibroblasts promote the stemness and progression of renal cell carcinoma via exosomal miR-181d-5p
Source: Cell Death Discov. 2022 Nov 1;8:439. doi: 10.1038/s41420-022-01219-7 (PMC9626570; doi:10.1038/s41420-022-01219-7)
Supplement: Supplementary file 2 — Supplementary Table 1 [file 41420_2022_1219_MOESM2_ESM.docx]

**Supplementary Table 1.** Correlation of α-SMA and CD105 expression to clinicopathologic characteristics in RCC patients.

| **Characteristics** | **Cases** | **α-SMA**  **IHC score *P* value^a^**  **(Mean ± s.d.)** | | **CD105**  **IHC score *P* value^a^**  **(Mean ± s.d.)** | |
| --- | --- | --- | --- | --- | --- |
|  | 141 |  |  |  |  |
| *Gender*  Male  Female | 95  46 | 4.774 ± 2.470  4.717 ± 2.363 | *0.8978* | 6.353 ± 3.099  5.815 ± 2.745 | *0.3186* |
| *Age*  >60  ≤60 | 65  76 | 4.492 ± 2.450  4.980 ± 2.401 | *0.2354* | 5.808 ± 3.139  6.493 ± 2.786 | *0.1754* |
| *Tumor grading^b^*  I-II  III-IV | 111  30 | 4.423 ± 2.142  5.983 ± 3.010 | ***0.0016*** | 5.874 ± 2.888  7.300 ± 3.139 | ***0.0199*** |
| *Distant metastasis*^b^  M0  M1 | 135  6 | 4.681 ± 2.402  6.417 ± 2.616 | *0.0867* | 6.144 ± 3.012  6.917 ± 2.518 | *0.5377* |
|  |  |  |  |  |  |

^a^ **P** values were derived using two-tailed Student’s t-test to compare values for the two parameters in each category.

^b^ Tumor grading and metastasis were classified according to the WHO Classification for staging renal carcinoma*.*
