# Supplementary material for: Cancer-associated fibroblasts promote the stemness and progression of renal cell carcinoma via exosomal miR-181d-5p
Source: Cell Death Discov. 2022 Nov 1;8:439. doi: 10.1038/s41420-022-01219-7 (PMC9626570; doi:10.1038/s41420-022-01219-7)
Supplement: Supplementary file 4 — Supplementary Table 3 [file 41420_2022_1219_MOESM4_ESM.docx]

| **Locus** | **primer** |
| --- | --- |
| F-preMIR-181d | ACATTCATTGTTGTCGGTGGGT |
| R-preMIR-181d | GTGACATTCATCCCCCGGTGGGT |
| F-preMIR-183 | ATGGCACTGGTAGAATTCACT |
| R-preMIR-183 | TATGGCCCTTCGGTAATTCACT |
| F-preMIR-561 | TCAAGGATCTTAAACTTTGCC |
| R-preMIR-561 | TTCAAGGATCTTAAACTTTGCC |
| F-preMIR-584 | TATGGTTTGCCTGGGACTGAG |
| R-preMIR-584 | GCCTGGTTGGCCTGGAACTGAC |
| F-preMIR-146a | GAGAACTGAATTCCATGGGTT |
| R-preMIR-146a | AAGAACTGAATTTCAGAGGTCT |
| RT-miR-181d-5p | CTCAACTGGTGTCGTGGAGTCGGCAATTCAGTTGAGACCCACCG |
| Q-miR-181d-5p | ACACTCCAGCTGGGAACATTCATTGTTGTC |
| RT-miR-1307-3p | CTCAACTGGTGTCGTGGAGTCGGCAATTCAGTTGAGCACGACCG |
| Q-miR-1307-3p | ACACTCCAGCTGGGACTCGGCGTGGCGTCG |
| RT-miR-183-5p | CTCAACTGGTGTCGTGGAGTCGGCAATTCAGTTGAGAGTGAATT |
| Q-miR-183-5p | ACACTCCAGCTGGGTATGGCACTGGTAGAA |
| RT-miR-561-5p | CTCAACTGGTGTCGTGGAGTCGGCAATTCAGTTGAGGGCAAAGT |
| Q-miR-561-5p | ACACTCCAGCTGGGATCAAGGATCTTAAAC |
| RT-miR-584-5p | CTCAACTGGTGTCGTGGAGTCGGCAATTCAGTTGAGCTCAGTCC |
| Q-miR-584-5p | ACACTCCAGCTGGGTTATGGTTTGCCTGGGA |
| RT-miR-330-3p | CTCAACTGGTGTCGTGGAGTCGGCAATTCAGTTGAGTCTCTGCA |
| Q-miR-330-3p | ACACTCCAGCTGGGGCAAAGCACACGGCCT |
| RT-miR-135a-5p | CTCAACTGGTGTCGTGGAGTCGGCAATTCAGTTGAGTCACATAG |
| Q-miR-135a-5p | ACACTCCAGCTGGGTATGGCTTTTTATTCC |
| RT-miR-146a-3p | CTCAACTGGTGTCGTGGAGTCGGCAATTCAGTTGAGCTGAAGAA |
| Q-miR-146a-3p | ACACTCCAGCTGGGCCTCTGAAATTCAGTT |
| RT-miR-96-5p | CTCAACTGGTGTCGTGGAGTCGGCAATTCAGTTGAGAGCAAAAA |
| Q-miR-96-5p | ACACTCCAGCTGGGTTTGGCACTAGCACAT |
| RT-miR-146a-5p | CTCAACTGGTGTCGTGGAGTCGGCAATTCAGTTGAGAACCCATG |
| Q-miR-146a-5p | ACACTCCAGCTGGGTGAGAACTGAATTCCA |
| RT-U6 | CTCAACTGGTGTCGTGGAGTCGGCAATTCAGTTGAGAACGCTTC |
| Q-U6 | ACACTCCAGCTGGGACGCAAATTCGTGAAG |
| Unifield-Q | TGGTGTCGTGGAGTCGGCAA |
| F-RNF43 | ACTCTGTGGTCAACTGCACG |
| R-RNF43 | CACTAGGCTGCATGTCCACT |
| F-OCT4 | CAAAGCAGAAACCCTCGTGC |
| R-OCT4 | TGATCTGCTGCAGTGTGGG |
| F-ALDH1A1 | ATCAAAGAAGCTGCCGGGAA |
| R-ALDH1A1 | GCATTGTCCAAGTCGGCATC |
| F-actin | CTACCTCATGAAGATCCTCACCGA |
| R-actin | TTCTCCTTAATGTCACGCACGATT |

**Supplementary Table 3. qRT-PCR primer sequences**
