# Supplementary material for: Cancer-associated fibroblasts promote the stemness and progression of renal cell carcinoma via exosomal miR-181d-5p
Source: Cell Death Discov. 2022 Nov 1;8:439. doi: 10.1038/s41420-022-01219-7 (PMC9626570; doi:10.1038/s41420-022-01219-7)

Figure 2B

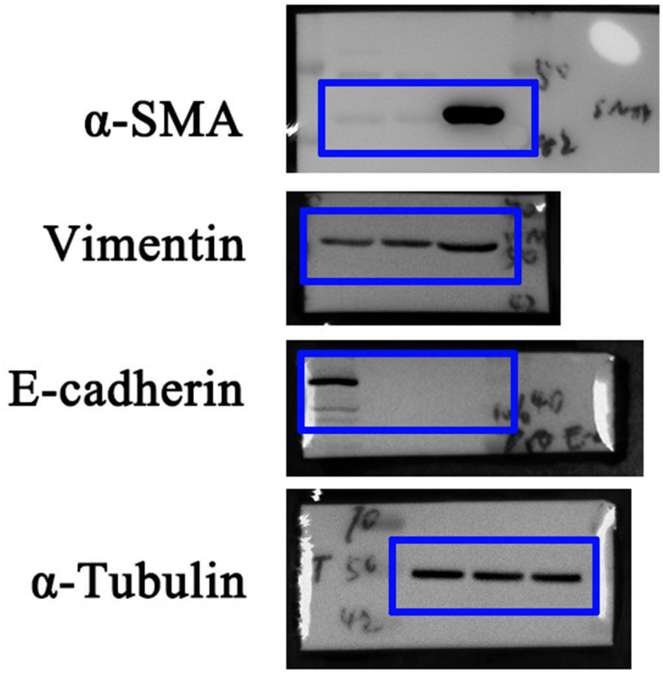

Figure 2G

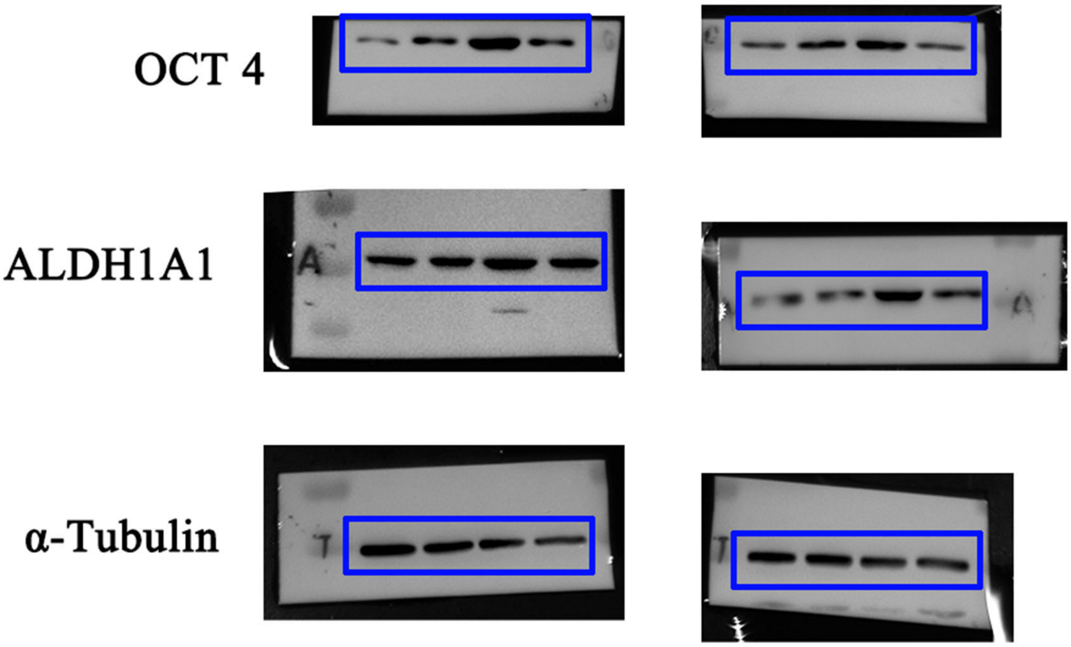

Figure 3B

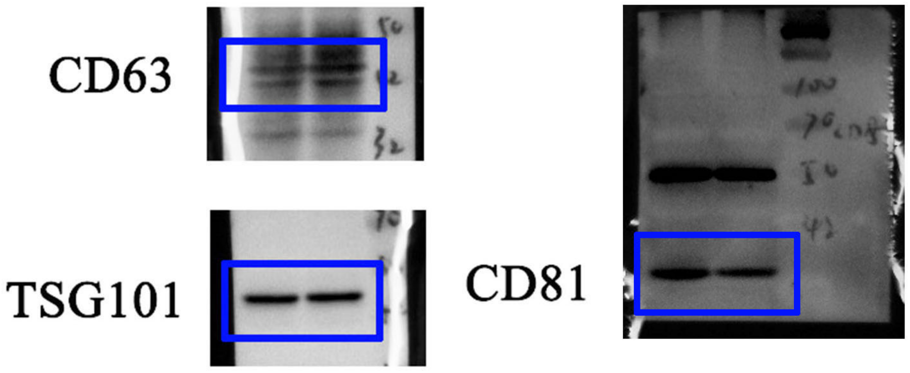

Figure 3G

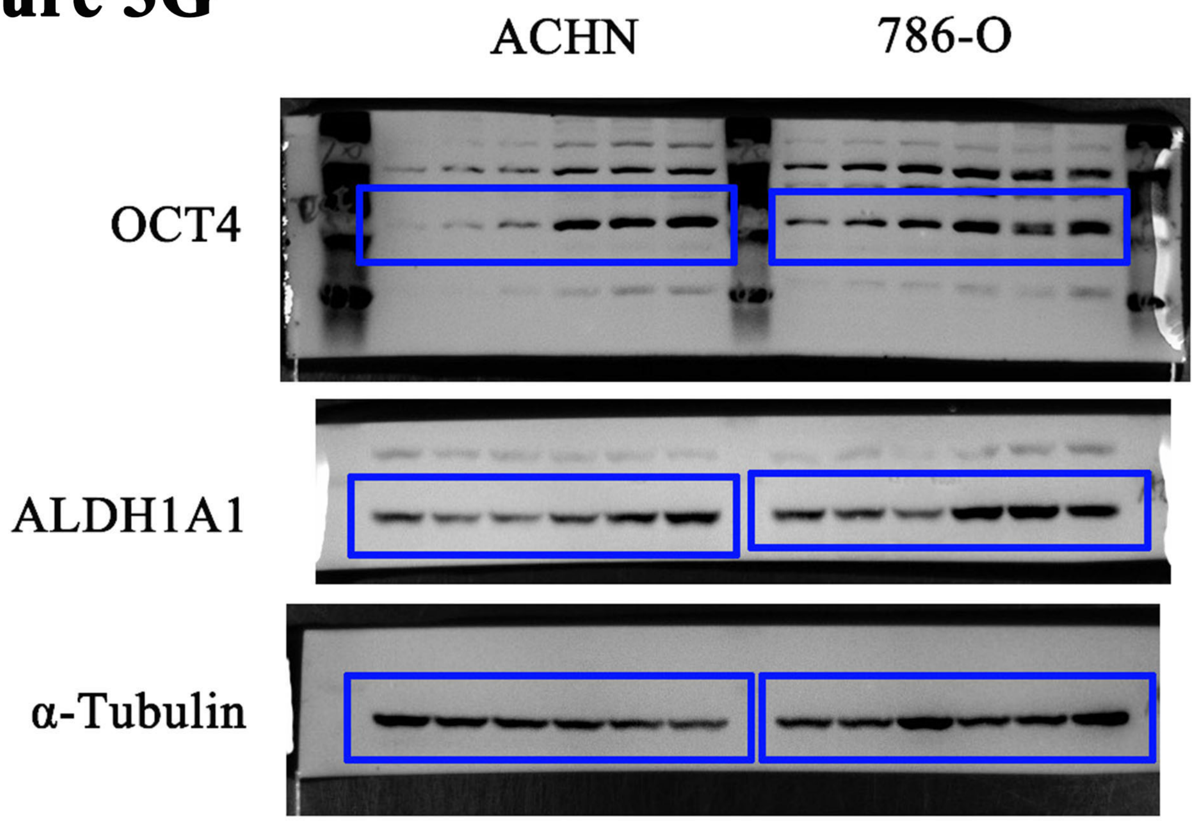

Figure 5E

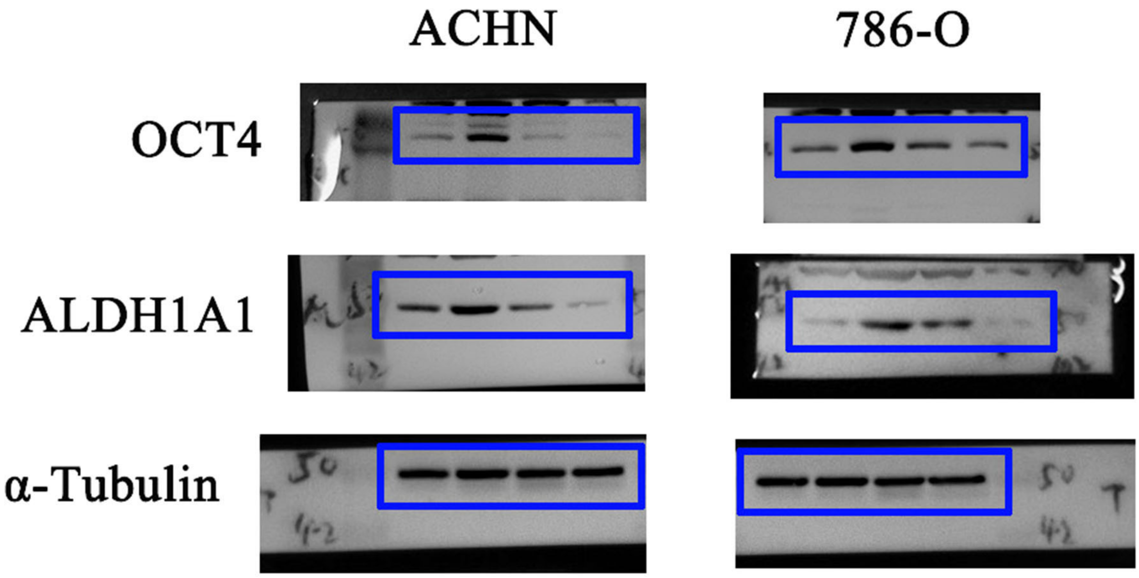

Figure 6F

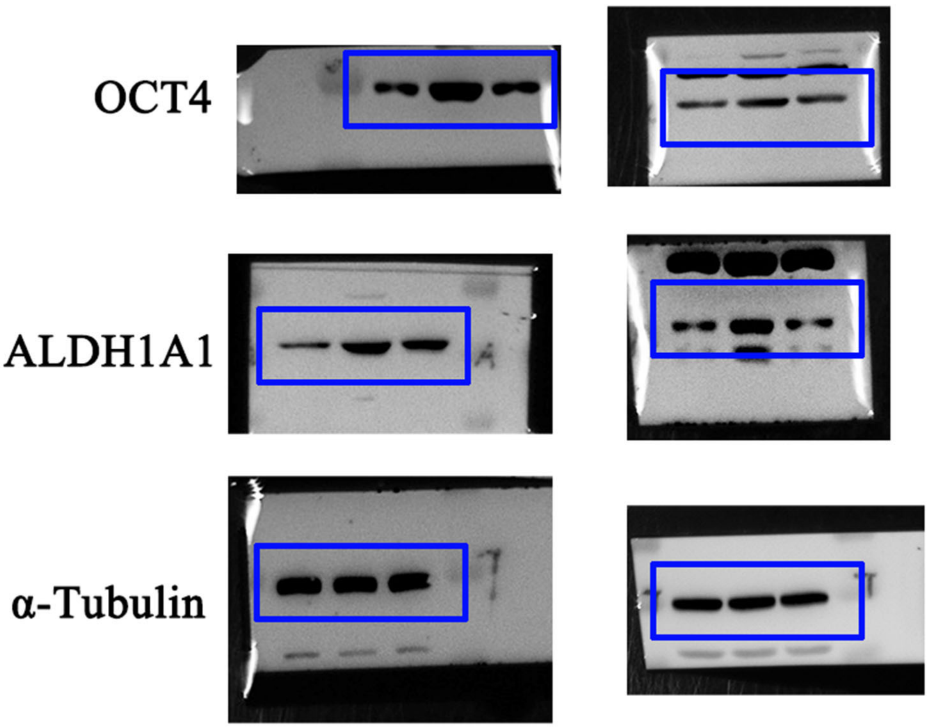

Figure 7C

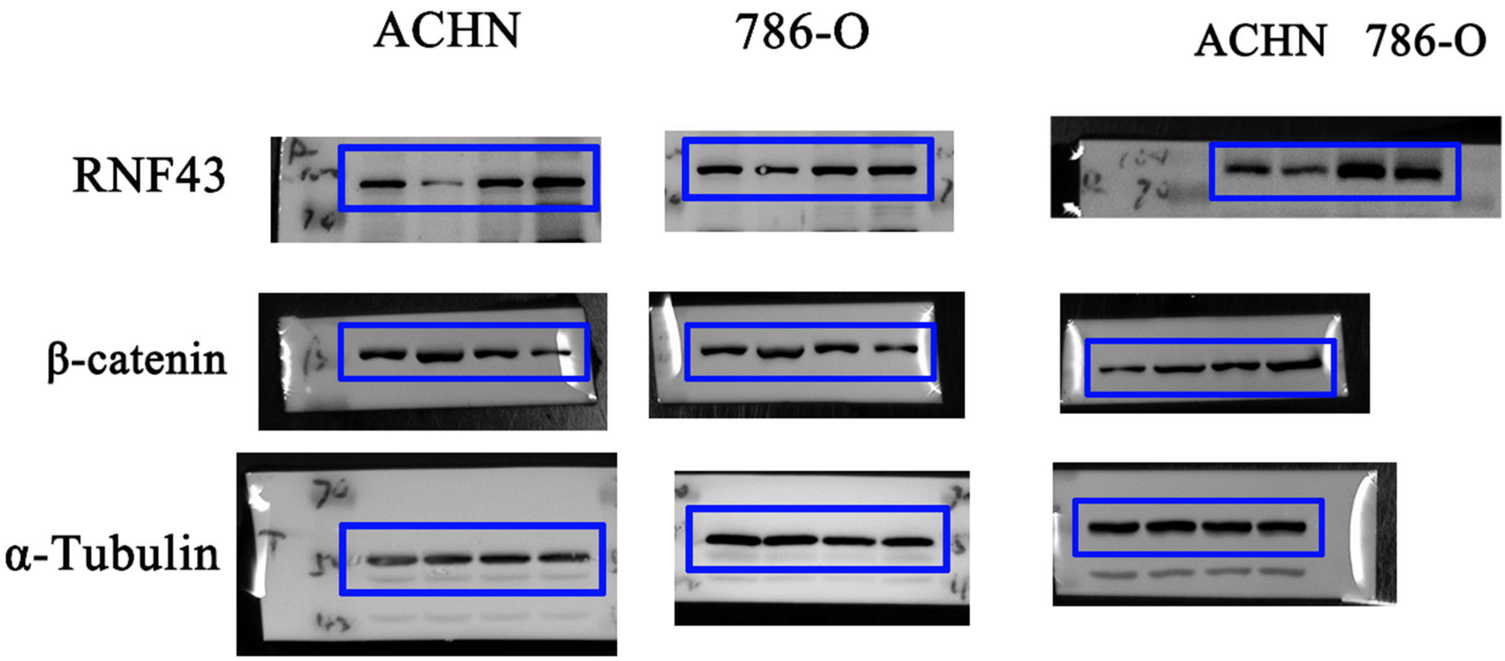

Figure 8E

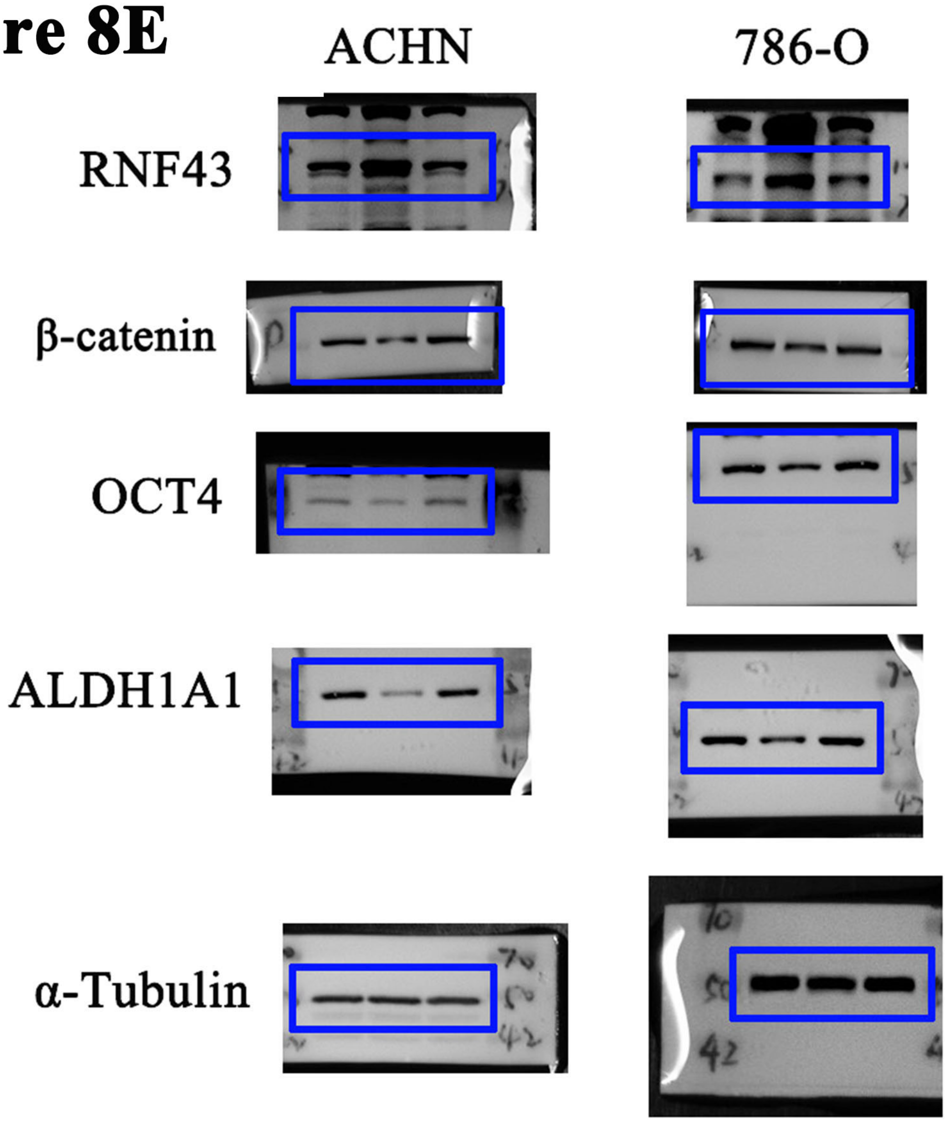

Figure 9E

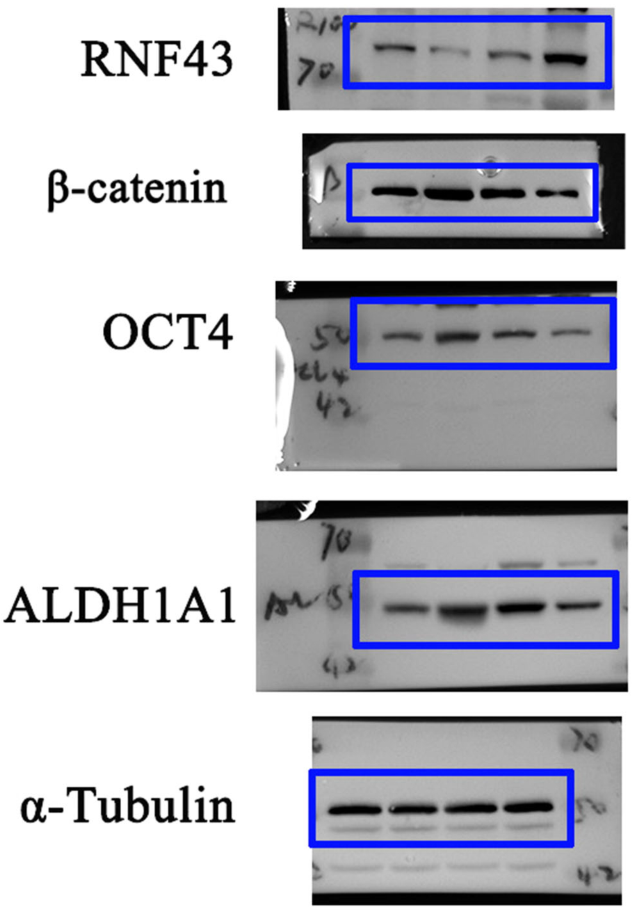

Figure 9J

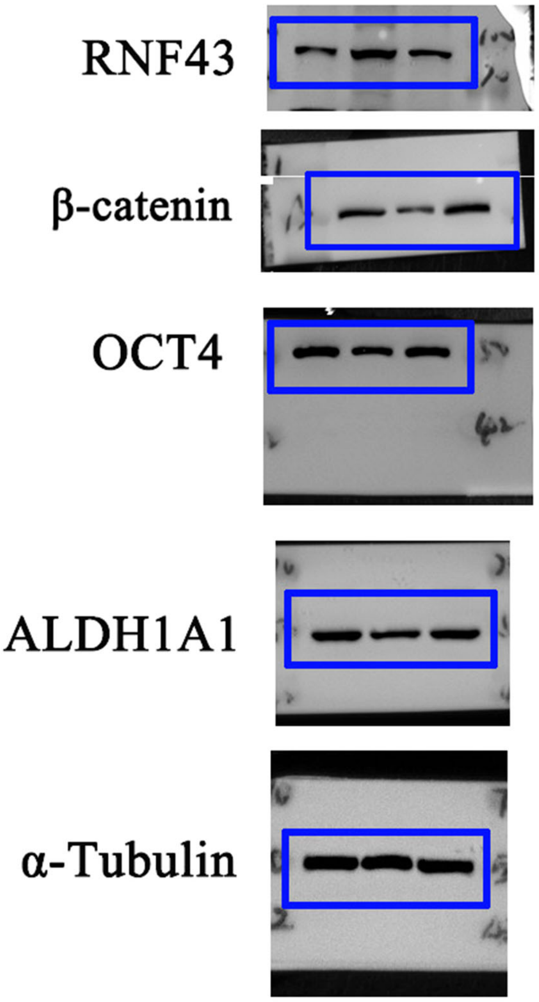

Supplementary Figure 3C

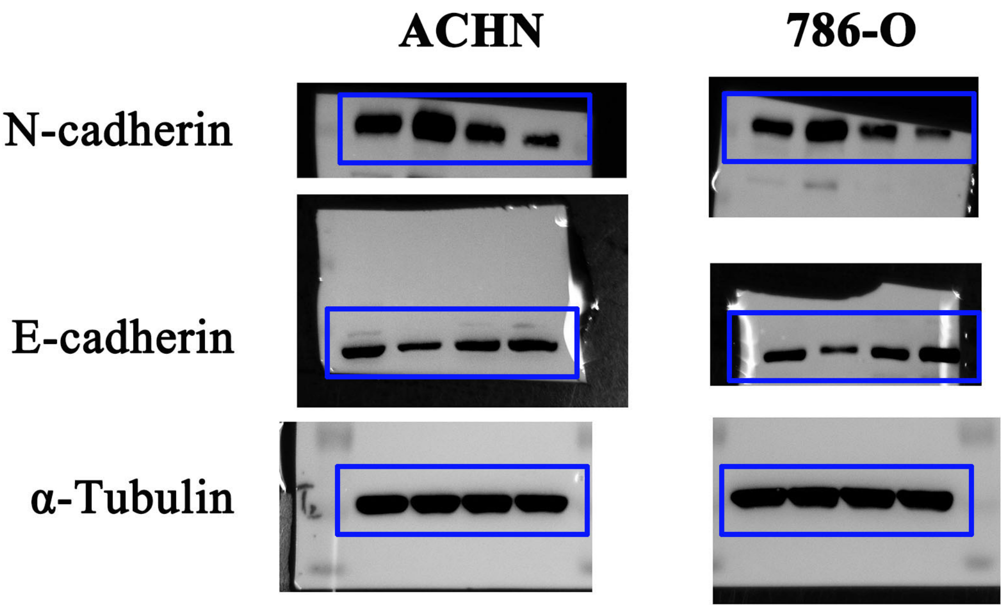

Supplementary Figure 3D

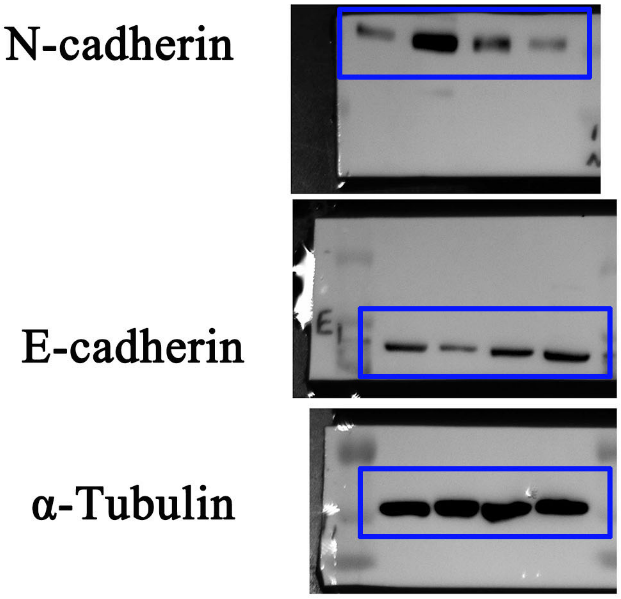

Supplement: Supplementary file 5 — Original data file [file 41420_2022_1219_MOESM5_ESM.pdf]
